# The Future of e-Learning in Medical Education: Current Trend and Future Opportunity

**DOI:** 10.3352/jeehp.2006.3.3

**Published:** 2006-09-12

**Authors:** Sara Kim

**Affiliations:** Department of Family Medicine and Department of Medical Education and Biomedical Informatics, School of Medicine, University of Washington, Box 356390 Seattle, WA 98195-7230, U.S.A.

**Keywords:** Education, Medical, Computer-Assisted Instruction, Learning

## Abstract

A wide range of e-learning modalities are widely integrated in medical education. However, some of the key questions related to the role of e-learning remain unanswered, such as (1) what is an effective approach to integrating technology into pre-clinical vs. clinical training?; (2) what evidence exists regarding the type and format of e-learning technology suitable for medical specialties and clinical settings?; (3) which design features are known to be effective in designing on-line patient simulation cases, tutorials, or clinical exams?; and (4) what guidelines exist for determining an appropriate blend of instructional strategies, including on-line learning, face-to-face instruction, and performance-based skill practices? Based on the existing literature and a variety of e-learning examples of synchronous learning tools and simulation technology, this paper addresses the following three questions: (1) what is the current trend of e-learning in medical education?; (2) what do we know about the effective use of e-learning?; and (3) what is the role of e-learning in facilitating newly emerging competency-based training? As e-learning continues to be widely integrated in training future physicians, it is critical that our efforts in conducting evaluative studies should target specific e-learning features that can best mediate intended learning goals and objectives. Without an evolving knowledge base on how best to design e-learning applications, the gap between what we know about technology use and how we deploy e-learning in training settings will continue to widen.

## INTRODUCTION

The direction of the future of e-learning in medical education is heavily influenced by three major trends: (1) the rapid adoption of emerging communication, simulation, and information technology in undergraduate, graduate, and continuing medical education; (2) a national call for competency-based, patient outcome-oriented training across the continuum of education; and (3) the rapidly changing health care environment including advances in the biomedical sciences as well as in the diagnoses and management of diseases, organization, financing, and delivery of health care services, and changes in the societal expectations [1, 2]. This paper provides an overview of the current state of e-learning in medical education and examines whether the current trend in e-learning can adequately serve the needs of medical education in the 21st century.

E-learning is defined as learning mediated by technology, such as the World Wide Web, intranet, and multi-media based computer applications [3, 4]. In recent years, the emergence of virtual universities promises a revolutionary approach to training medical doctors using cutting edge e-learning technologies. The International Virtual Medical School (IVIMEDS) (http://www.ivimeds.org/) and the Virtual Campus of the King's College of University of London (http://gktvc1.kcl.ac.uk/) are examples of e-learning training at the undergraduate, residency, and continuing professional levels [5]. The IVIMEDS consists of more than 30 partners in 15 countries, who agree to share (1) curriculum maps that link learning content and assessment, (2) learning resources including illustrations, video clips, animated diagrams, medical images, and (3) virtual patients that simulate authentic, high fidelity patient problems. The Virtual Campus of the King's College offers Web-based systems that provide learning and administrative support to medical, dental, and health sciences students. As more and more institutions are seeking cost-effective approaches to optimizing the capacity of e-learning in medical training, there is no doubt that these virtual universities will have an increasing appeal in the coming years.

As new technology is developed and deployed in learning settings at a rapid pace, the question that begs an answer is: what specific contributions can technology make towards improving the quality of medical education? Even with today's extensive and pervasive use of technology in medical education, some of the basic questions related to the role of e-learning remain unanswered [3, 6], such as:

1. What is an effective approach to integrating technology into pre-clinical vs. clinical training?
2. What evidence exists regarding the type and format of e-learning technology suitable for medical specialties and clinical settings?
3. Which design features are known to be effective in designing on-line patient simulation cases, tutorials, or clinical exams?
4. What guidelines exist for determining an appropriate blend of instructional strategies, including on-line learning, face-to-face instruction, and performance-based skill practices?

The central tenet of this paper is three fold. First, technology will continue to dominate the way we train future physicians. Second, there exists an untapped potential for synchronous delivery and simulation technology to help meet training needs in emerging competencies [7]. Third, we have an opportunity to build a knowledge base that can guide both practitioners and researchers in developing effective technology-based learning using evidence-based findings. This paper is organized around three main questions:

1. What is the current trend of e-learning in medical education?
2. What do we know about the effective use of e-learning?
3. What is the role of e-learning in facilitating newly emerging competency-based training?

## What is the Current Trend of e-Learning in Medical Education?

**Fig. 1:**
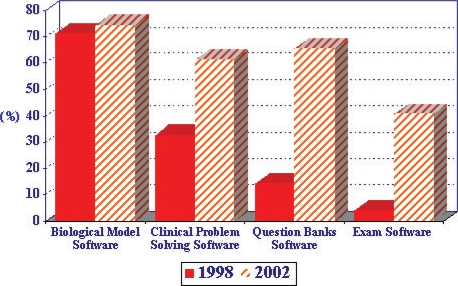
Comparison of percentages of 125 US medical schools reporting the use of eductional software program in basic sciences curriculum in 1998 and 2002.

**Fig. 2:**
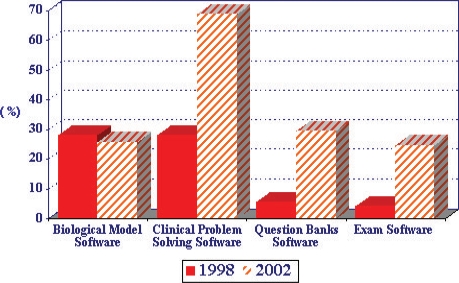
Comparison of percentages of 125 US medical schools reporting the use of eductional software program in clinical clerkships in 1998 and 2002.

The Liaison Committee on Medical Education in the U.S. conducts an annual survey of medical schools, in which several questions are asked about the use of technology in the medical curriculum [8]. One item asks whether educational software applications are used in the required basic sciences courses and clinical clerkships. Fig. 1 shows the percentage of medical schools that report the use of biological modeling software, clinical problem solving software, question banks software and exam software in their basic sciences courses. Data collected in 1998 are compared with 2002 survey results. As shown in Fig. 1, a greater proportion of the surveyed 125 US Medical Schools report that the use of educational software programs has increased in 2002 compared to 1998. Although to a smaller degree, Fig. 2 shows a similar trend in the percentage of schools reporting the increased use of educational software programs in their clinical clerkships.

At the CME (continuing medical education) level, Curran and Fleet [9] report that over one-year period between 2000 and 2001, the number of available CME Web sites increased from 96 to more than 200 Web sites. It is not clear the degree to which e-learning is integrated in residency training, but randomized control trials examining the effectiveness of Web-based learning on residents' knowledge and satisfaction attest to the value of e-learning in training residents [10, 11]. In addition, the recently mandated ACGME (Accreditation of Council for Graduate Medical Education) competencies call for an increased use of information technology for competency- based teaching and assessment (http://www.acgme.org/Outcome/).

At the national and international levels, a number of initiatives have emerged with the purpose of creating a digital repository of peer-reviewed electronic resources for public dissemination [3]. Some of the examples include: MedEd Portal by Association of American Medical Colleges (http://www.aamc.org/mededportal), End of Life/Palliative Education Resource Center by Medical College of Wisconsin (http://www.eperc.mcw.edu/), The Health Education Assets Library (HEAL, http://www.healcentral.org/), Multimedia Educational Resource for Learning and On-line Teaching (MERLOT, http://www.merlot.org), and Family Medicine Digital Resource Library (http://fmdrl.org). These initiatives recognize the need to create a mechanism for sharing quality e-learning resources across institutions and to reward the works of faculty and staff through peer reviewed processes.

## Technology is Here to Stay: Do We Know What Works?

**Fig. 3:**
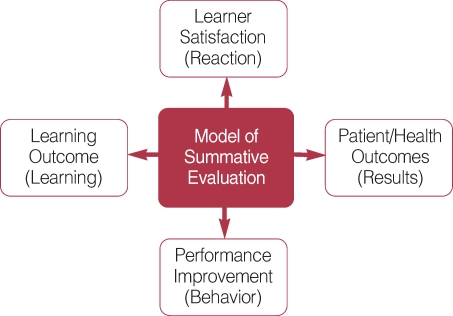
Kirkpatrick's model of summative evaluation.

In understanding the impact of e-learning on learning, Kirkpatrick's work has been cited as a useful model [12]. The model consists of four dimensions, including learner satisfaction, learning outcomes, performance improvement, and patient/ health outcomes (Fig. 3).

Learner satisfaction mainly encompasses participants' perceptions and satisfaction with learning objectives, content, format, and instructor's effectiveness. Learning outcomes include assessment of learners' knowledge, attitudes, and skills. Performance improvement targets changes in practice behaviors as a direct result of the newly acquired knowledge, attitudes, and skills. Lastly, patient and health outcomes include changes in patients' behaviors and health indicators as a result of trainees' improved practice patterns. For example, in a study by Curran and Fleet [9], 10 which examined CME-related e-learning studies, 81% of the reviewed studies included evaluation of learner's satisfaction, followed by 52% targeting learning outcomes and 7% evaluating student performance change in clinical practice. No studies included patient or health outcomes as part of the evaluation.

A number of systematic reviews have been conducted to examine the effectiveness of e-learning across the continuum of medical training covering medical students, residents, and practicing physicians. Appendix 1 summarizes results from the recently published studies [7, 9, 13-16]. Using Kirkpatrick's model as a guide for understanding the trend of studies involving e-learning in medical education, several conclusions can be drawn from the systematic reviews. First, most of the reported studies are descriptive in nature with little evaluative data. They tend to describe the development and implementation processes involving e-learning applications without concrete data to demonstrate the impact of e-learning on learning outcomes, trainees' behaviors, or patient outcomes. Second, most of the reported evaluation focuses on multiple-choice questions assessing trainees' knowledge using pre-and post-tests. Few studies examined other outcome measures to assess areas of competencies that are difficult to evaluate using multiple-choice questions, such as communication skills or decision-making skills. Third, reported data tend to focus on trainees' subjective impression, mostly their satisfaction with e-learning, rather than using validated outcome measures. Lastly, the majority of the studies showed weak or inappropriate study design with few randomized controlled trials. As a result of these trends in how e-learning applications are evaluated in medical education, the knowledge base for how to design effective e-learning systems for targeting trainees' satisfaction, learning, performance behavior, and patient outcomes remains underdeveloped.

One example of a novel approach to incorporating Kirkpatrick's model in assessing an e-learning application involves the Case for Change tool developed at the University of Washington (http://fammed.washington.edu/predoctoral/clerkship/casedemo.html). The purpose of the Case for Change is to teach third-year medical students how to conduct a patient-centered communication targeting a patient's behavioral change. This Web-based tool is required of all students to complete during their 6-weeks of Family Medicine clerkship rotation. These rotations take place at 24 training sites in the five-state region (Washington, Wyoming, Alaska, Montana, Idaho). The main components of Case for Change include: (1) video cases that simulate a provider-patient interaction using a scenario of reducing risky sexual behavior; (2) free-text input by students what they would say to the patient after viewing the video segments; and (3) multiple choice options selected by students that best match their free-text inputs. Multiple video segments are available to students that are associated with key stages of patient-centered behavioral change discussions, including building rapport, eliciting patient concerns, negotiating agenda, determining risk patterns, etc. Our evaluation involved coding of students' free-text responses and scoring of their multiple choice options using the expert's approach as the gold standard. Four categories of coding were used in analyzing students' free-text responses:

1. Pre-determined Desired Themes: Students' input that matched instructor's learning goals and objectives for individual video segment.
2. Student-initiated Acceptable Themes: Positive comments offered by students that are indirectly related to learning goals and objectives.
3. Pre-determined Undesired Themes: Students' input contradictory to learning goals and objectives.
4. Student-initiated Unacceptable Themes: Comments that are not acceptable, such as comments that display unprofessional behaviors on a student's part.

For example, under negotiating agenda, students' comments pertaining to summarizing the patient's concern, mentioning time constraint in the encounter, and helping patient prioritize agenda were considered to be pre-determined desired theme. On the other hand, comments that suggested physician-directed agenda were coded as pre-determined undesired theme. Our preliminary evaluation based on 112 students' performance shows that in most categories of the case segment, fewer students received correct scores on their free-text responses compared to their multiple choice options, with the exception of building rapport (Table 1). Particularly, these differences were notable with statistical significance in the categories of eliciting patient concerns, determining risk patterns, and behavior change discussions.

From this evaluation, we identified several areas in students' behaviors that need explicit teaching in the future. These areas included students' tendencies to dive into premature diagnostic questioning, to ask for a symptom list before eliciting a patient's concerns, determining the agenda without the patient's input, and using close-ended statements of diagnostic nature. Our future evaluation will attempt to correlate student's performance in Case for Change with faculty's observation of students interacting with patients during their clerkship rotations.

## What is the Role of Technology in New Competency-Based Training?

Training of physicians in the 21st century requires a new focus on emerging competencies. With the ACGME's (Accreditation Council of Graduate Medical Education) mandate required of all U.S. residency training programs, residents have to demonstrate their competencies in six core areas, including: (1) patient care, (2) medical knowledge, (3) practice-based learning and improvement, (4) interpersonal and communication skills, (5) professionalism, and (6) systems-based practice. There is an increasing call at the national level to (1) restructure the undergraduate and continuing medical education using the residency competencies [17]; and (2) to link competency-based training with patient outcomes [1].

So the question is: what is the role of technology in teaching and assessing competencies across undergraduate, graduate, and continuing medical education. How do we adapt the current e-learning applications for targeting the following competency skills in future physicians:

- Patient-centered communication skills
- Competency in providing culturally sensitive care
- Exhibiting professionalism in all aspects of a physician's life
- Exercising evidence-based decision making
- Patient safety/medical error reduction
- Inter-professional team care
- Life-long learning
- Continuous self-assessment
- Improving practice performance
- Evidence-based critical thinking and clinical reasoning

There are three e-learning modalities that promise a great potential for innovative training in the future. These modalities include: (1) simulation technology; (2) synchronous learning delivery; and (3) Web-based or videoconferencing for standardized patient-based training.

### Simulation Technology

The state of the art simulation centers exist in many U.S. medical institutions. These centers not only support performance- based training of surgery and anesthesiology trainees, but also internists and family physicians who are required to demonstrate a wide range of procedural skills. These skills encompass difficult airway management, cardiac life support, bronchoscopy, endoscopy, and suturing, etc. Goals of the simulation centers are to provide trainees with multiple opportunities to practice a wide range of procedural skills and to reduce preventable medical errors, thereby ensuring patient safety.

Some of the notable centers include: (1) National Capital Area Medical Simulation Center (http://simcen.usuhs.mil/home.html); (2) Harvard Center for Medical Simulation (http://www.harvardmedsim.org/); (3) Peter Winter Institute for Simulation, Education, Research at University of Pittsburgh (http://www.wiser.pitt.edu/); (4) Bristol Simulation Center (http://www.bris.ac.uk/Depts/BMSC/); (5) The Simulation Group at Massachusetts General Hospital (http://www.thesimgroup.org/); and (6) SimSuite from Medical Simulation Corporation (http://www.medsimulation.com/education_system/centers.asp). Typically, these centers use full mannequins or models connected to various display units that guide the trainees' performance during simulation sessions. Evaluating trainees' skills are usually conducted via observation by faculty members, who complete checklist forms for assessing trainees' psychomotor and technical skills. Depending on the scenarios developed for trainees' interactions with simulators, evaluation can also include trainees' skills in patient assessment, management of critical events, communication, and interpersonal relationship. Although expensive and required of highly skilled personnel to operate, simulation technologies will certainly be on the leading edge of e-learning applications in medical education. These technologies can serve as effective modalities for delivering competency-training in future physicians, which is difficult to deliver using conventional teaching methods.

### Synchronous Learning Delivery

The advancement in technology and access to broadband connectivity from remote training sites have made a synchronous learning delivery a possibility. The synchronous learning modality, such as Webcast, consists of a live video/audio broadcast of training sessions and archival of training materials for later access by participants. Many benefits include: (a) connecting learners from distant sites to live training sessions; (b) creating opportunities for trainers and participants to interact in real time; (c) fostering peer-to-peer feedback; (d) interacting with learning resources such as lecture notes or simulated cases; and (e) accessing training materials for self-paced review. The product developed by Macromedia, Breeze, offers one example of how a synchronous e-learning technology may be used in medical education (see http://www.adobe.com/products/breeze/for demonstration of system features). Breeze integrates a wide range of features for providing both instructors and learners a high level of flexibility. Instructors and learners can view each other via video streaming during live lectures, instructors can create on the fly on-line quizzes or surveys for learners to complete, and learning resources, such as Powerpoint slides and lecture notes, can be archived for students' self-paced learning. The entire Breeze session can be video or audio taped and archived for later access by learners as well. At University of Washington, third year students complete their clerkship at remote training sites in a five-state region that encompasses three-time zones. Breeze can be used to connect students or faculty members at clerkship sites to receive didactic sessions on commonly seen conditions in outpatient settings and to discuss interesting or rare cases that may not be frequently encountered at other sites.

### Web-based/Videoconferencing of Standardized Patients

The use of standardized patients has been an integral part of medical education for both teaching and assessment purposes. In recent years, Web-based OSCEs (Objective Structured Clinical Exams) and video technology have been piloted to test whether performance-based skills, such as decision making or error disclosure skills, can be taught and evaluated. One study by Clever, et al. [18] examined whether standardized patients (SPs) in Philadelphia can accurately evaluate informed decision making skills by an orthopedic surgeon connected via videoconferencing technology in Chicago. They concluded that SP-physician interaction was feasible in long-distance assessment of a variety of learners. Similarly, Chan, et al. [19] studied how effectively standardized patients were able to rate a surgeon's skills in disclosing medical errors. In this study, standardized patients were located in Toronto, Canada with surgeons based in St. Louis, Missouri, U.S.A. They concluded that videoconferencing was effective in assessing a physician's communication skills. In addition, a pilot study showed that medical students whose skills were assessed by SPs via the Web OSCEs, compared to students who interacted face-to-face with SPs, performed equally well in the category of physical exam and information giving skills [20].

## CONCLUSION

This paper presented a general overview of the current trend in e-learning with a focus on the U.S. medical education, main findings from the recent systematic reviews of studies involving e-learning that show many gaps in the way the effectiveness of e-learning is being examined, and a review of emerging technologies that have potentials for meeting new requirements for competency-based training. The paper also provided examples of emerging consortiums of institutions that have created common e-learning experiences for their medical trainees. As e-learning continues to be widely integrated in the training of future physicians, it is critical that our efforts in conducting evaluative studies should target specific e-learning features that can best mediate intended learning goals and objectives. Without an evolving knowledge base on how best to design e-learning applications, the gap between what we know about technology use and how we deploy e-learning in training settings will continue to widen.

## Figures and Tables

**Table 1:**
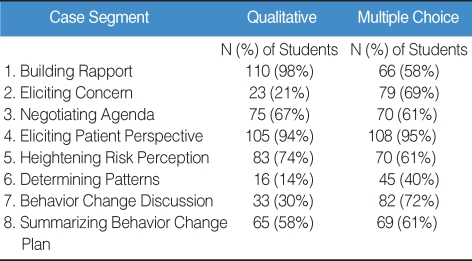
Number and % of Students with Correct Scores on Qualitative Comments and Multiple-Choice Questions

## Appendix 1

**Table:** Summary reviews of computer and web-based educational systems

Author/Year	Focus of Review	Number of Studies	Period Covered	Discipline/Specialty	Main Results & Implications
Adler & Johnson, 2000[13]	Provide a general overview of literature of computer-aided instruction (CAI) in medical education.	1,071	1966-1998	Medicine, Education	Most studies report demonstration projects without evaluative data. More studies are needed in CAI-to-CAI comparative studies rather than CAI-to-Non-CAI studies. Economic analyses associated with applications and technologies are needed.A greater knowledge base needed for understanding how to integrate CAI into a larger medical curriculum and how to evaluate CAI to understand its effectiveness in different learning environments involving different students.
Chumley-Jones, Dobbie and Alford, 2002[14]	Identify aspects of Web-based learning that have been studied.Describe evaluation strategies used in the reviewed studies.	76	1966-2002	Medicine, Dental, Nursing	The majority of studies were descriptive in nature with no evaluative data. Descriptive studies tended to report learners' satisfaction with learning tools.Among studies reporting data, the use of pre- & post- knowledge test using multiple choice question format was the most prevalent method.Only one study described direct and indirect costs associated with Web-based vs. text-based learning.Areas of unique contribution of Web-based learning in training of health professionals need to be more clearly defined.
Letterie, 2003[15]	To assess the quality of evidence for implementing computer-assisted instruction.	210	1988-2000	Medicine	Most studies were descriptive in nature. Studies positively endorsed featured technology without measure of effectiveness.The most widely used assessment measure included pre- and post-tests of knowledge.Few studies compared computer-assisted instruction with different learning modalities.
Lau and Bates, 2004[7]	To examine types and content of e-learning technology in undergraduate medical education.	50	1997-2002	Medicine(undergrad)	The majority of studies descriptive in nature.Lack of study design makes it difficult to judge the quality of descriptive reports.The majority of evaluation measures included user satisfaction, actual usage, subjective feedback, and student performance.
Curran and Fleet, 2005[9]	To examine the nature and characteristics of Web-based continuing medical education evaluative outcomes.	86	1966-2003	Medicine(continuing medical education)	The majority of evaluative research is based on participant satisfaction data.Lack of systematic evidence that suggests that Web-based CME enhances clinical practice performance or patient/health outcomes.
Issenberg, McGaghie, Petrusa, Gordon, and Scalese, 2005[16]	Review and synthesize existing evidence in the literature of features and uses of high-fidelity medical simulations that lead to effective learning.	109	1966-2003	Medicine	Among the target features, 47% of the reviewed articles reported that feedback is the most important feature of simulation-based medical education; 39% identified repetitive practice as a key feature involving the use of high-fidelity simulations, 25% cited the need to integrate simulation in the curriculum is an essential feature, and 14% highlighted the range of task difficulty as an important variable. Less than 10% of the reviewed studies cited the following features as important factors for simulations: multiple learning strategies, capture clinical variation, controlled environment, individualized learning, defined outcomes, and simulator validity correlated with learning.
